# Supplementary material for: Participant experiences of a low-energy total diet replacement programme: A descriptive qualitative study
Source: PLoS One. 2020 Sep 8;15(9):e0238645. doi: 10.1371/journal.pone.0238645 (PMC7478843; doi:10.1371/journal.pone.0238645)
Supplement: S1 Checklist — (DOCX) [file pone.0238645.s001.docx]

|  | Manuscript page and line number |
| --- | --- |
| **Domain 1: Research team and reflexivity** | |
| **Personal characteristics** |  |
| Which authors conducted the interview or focus group? | P7 L110-113 |
| What were the researchers credentials? | P7 L110-113 |
| What was their occupation at the time of the study? | P7 L110-113 |
| Was the researcher male or female? | P7 L110-113 |
| What experience or training did the researcher have? | P1 L117-118 |
| **Relationship with participants** |  |
| Was a relationship established prior to study commencement? | P7 L113-117 |
| What did the participants know about the researcher? | P7 L105-107 & L113-117 |
| What characteristics were reported about the interviewer/facilitator? | P8 128-129 |
| **Domain 2: study design** | |
| **Theoretical framework** |  |
| What methodical orientation was stated to underpin the study? | P8 139-143 |
| **Participant selection** |  |
| How were participants selected? | P6 L89-96 |
| How were participants approached? | P6 L94-95 |
| How many participants were in the study? | P6 L96-99 |
| How many people refused to participate or dropped out? Reasons? | P6 L96-99 |
| **Setting** |  |
| Where was the data collected? | P7 L118-120 |
| Was anyone else present besides the participants and researchers? | P7 L118-120 |
| What are the important characteristics of the sample? | P9 171-173 & P4 174-179 & Table 1 |
| **Data collection** |  |
| Were question, prompts, guides provided by the authors? Was it pilot tested? | P4/5 L124-129 |
| Were repeat interviews carried out? If yes, how many? |  |
| Did the research use audio or visual recording to collect the data? | P7 L118-120 |
| Were field nots made during and/or after the interview or focus groups? | P7 L118-120 |
| What was the duration of the interviews or focus groups? | P7 123-124 |
| Was data saturation discussed? | P7&8 L100-103 & 1L30-132 |
| Were transcripts returned to participants for comments and/or correction? | P7 L120-122 |
| **Domain 3: analysis and findings** | |
| How many coders coded the data? | P9 L158-161 |
| Did authors provide a description of the coding tree? | L162-168 |
| Were themes identified in advance or derived from the data? | L158-160 |
| What software, if applicable was used to manage the data? | L167-168 |
| Did participants provide feedback on the findings? |  |
| Were participant quotations presented to illustrate the themes/findings?  Was each quotation identified? | yes |
| Was there consistency between the data presented and the findings? | Yes |
| Is there a description of diverse cases or discussion of minor themes? | N/A |
