# Supplementary material for: Participant experiences of a low-energy total diet replacement programme: A descriptive qualitative study
Source: PLoS One. 2020 Sep 8;15(9):e0238645. doi: 10.1371/journal.pone.0238645 (PMC7478843; doi:10.1371/journal.pone.0238645)
Supplement: S1 Material — (PDF) [file pone.0238645.s002.pdf]

DROPLET Study Interview Guide

Why did you decide to take part in the DROPLET study?

What did you expect from taking part in the DROPLET study?

How did you feel when you were allocated to the treatment group?

-Before being randomised, did you have a preference to which group you were allocated?

Can you describe your experience of taking part in the DROPLET Study?

- What did you think of the person, nature, frequency and length of the treatment you were allocated?
- What were you told to do as part of the allocated treatment programme?
- What support were you offered?
- Was the programme what you expected it to be like?

Were you able to stick to the programme?

What aspects of the programme made it easy/difficult to follow?

How did you cope with difficult moments?

What did you like/dislike about the treatment?

How was this experience similar or different from past weight loss experiences?

Are you happy with the outcomes?

Do you plan to continue with the programme you were allocated? Why?
